# Total Internal Reflection Accounts for the Bright Color of the Saharan Silver Ant

**DOI:** 10.1371/journal.pone.0152325

**Published:** 2016-04-13

**Authors:** Quentin Willot, Priscilla Simonis, Jean-Pol Vigneron, Serge Aron

**Affiliations:** 1 Evolutionary Biology & Ecology, Université Libre de Bruxelles, Brussels, Belgium; 2 Photonic of living Organisms Group, Research Center in Physics of Matter and Radiation (PMR), University of Namur, Namur, Belgium; University of Akron, UNITED STATES

## Abstract

The Saharan silver ant *Cataglyphis bombycina* is one of the terrestrial living organisms best adapted to tolerate high temperatures. It has recently been shown that the hairs covering the ant’s dorsal body part are responsible for its silvery appearance. The hairs have a triangular cross-section with two corrugated surfaces allowing a high optical reflection in the visible and near-infrared (NIR) range of the spectrum while maximizing heat emissivity in the mid-infrared (MIR). Those two effects account for remarkable thermoregulatory properties, enabling the ant to maintain a lower thermal steady state and to cope with the high temperature of its natural habitat. In this paper, we further investigate how geometrical optical and high reflection properties account for the bright silver color of *C*. *bombycina*. Using optical ray-tracing models and attenuated total reflection (ATR) experiments, we show that, for a large range of incidence angles, total internal reflection (TIR) conditions are satisfied on the basal face of each hair for light entering and exiting through its upper faces. The reflection properties of the hairs are further enhanced by the presence of the corrugated surface, giving them an almost total specular reflectance for most incidence angles. We also show that hairs provide an almost 10-fold increase in light reflection, and we confirm experimentally that they are responsible for a lower internal body temperature under incident sunlight. Overall, this study improves our understanding of the optical mechanisms responsible for the silver color of *C*. *bombycina* and the remarkable thermoregulatory properties of the hair coat covering the ant’s body.

## Introduction

*Cataglyphis bombycina*, the”silver ant” of the Sahara, the Sinai and the deserts of the Arabian Peninsula, is famous for its ability to withstand extremely high temperatures [1,2]. Workers come out from the nest during the hottest midday period, when temperatures exceed 50°C, to scavenge corpses of heat-stricken animals. By restricting foraging activity to the hottest period of the day, the ants minimize the chances of encountering their most frequent predator—a lizard that ceases all activities when the temperature becomes unbearable. This, however, requires that the ants themselves are effectively protected against excessive temperatures. Workers strictly limit the time of exposure to the light from the high sun and the heat radiated by the hot sand [2]. They are equipped with legs much longer than other ants relative to their body shape, allowing them to keep their body at a greater distance from the hot surface, and to run much faster reducing the duration of their sorties while maximizing cooling by convection [1,3,4]. In contrast to other organisms, workers produce heat-shock proteins before emerging from the nest, thereby avoiding a slow adaptation to the sudden heat exposure [5,6].

**Fig 1:**
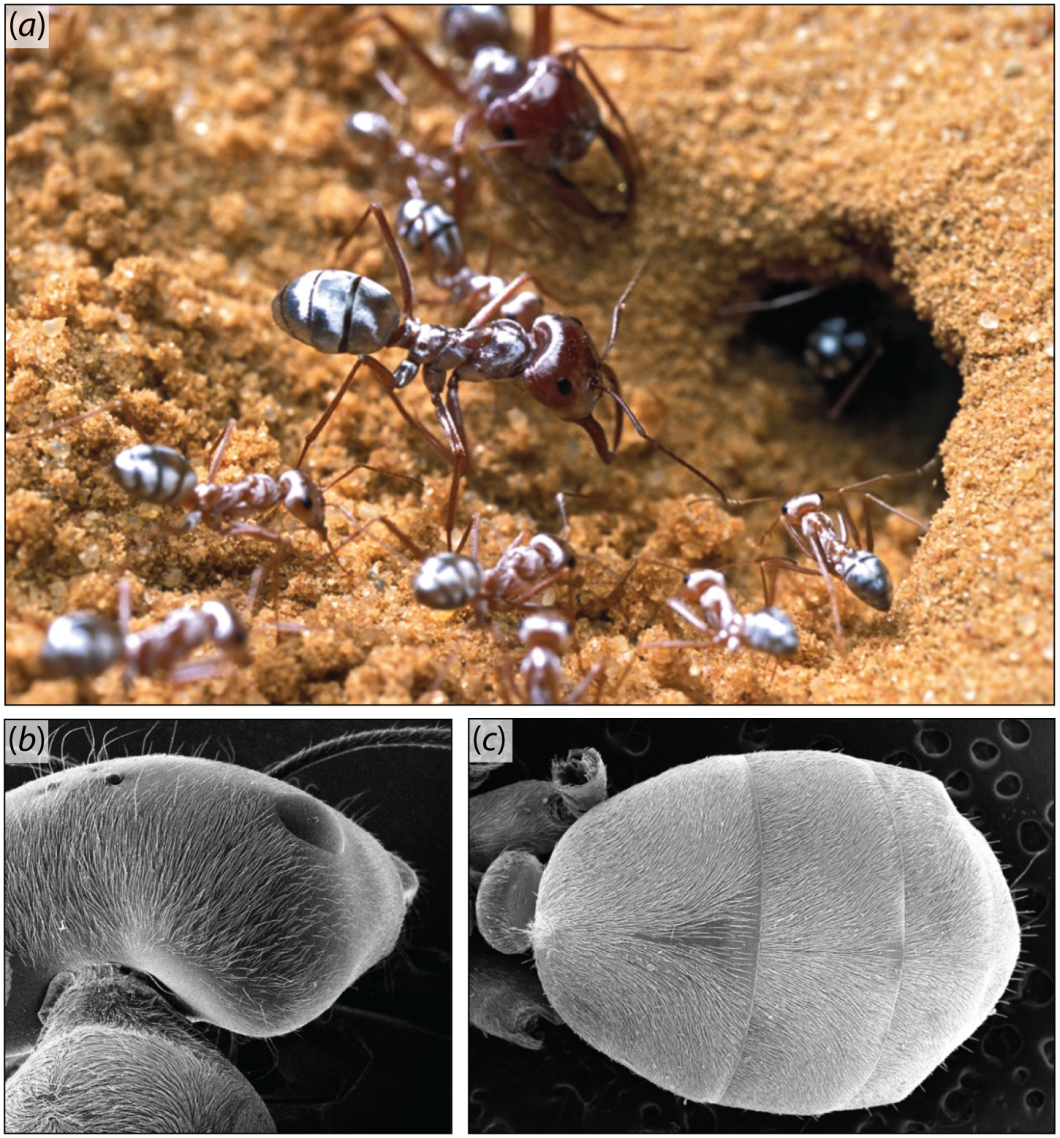
The silver ant *Cataglyphis bombycina*. Colonies contain a caste of spindly workers and a caste of soldiers with large heads and saber-shaped mandibles. (a) In full sunlight, workers and soldiers show a metallic sheen that justifies the vernacular name of the species. Photo copyright: P. Landmann. (b,c) The hairs covering the dorsal side of the workers’ head, thorax (b) and abdomen (c) follow the cuticle’s curvatures.

Recently, it has been shown that silver ants have evolved remarkable thermoregulatory solutions to cope with the thermally stressful conditions of their natural environment [7]. The dorsal side of the workers’ head, thorax and abdomen is covered with a dense array of triangularly shaped hairs that are responsible for the ants silvery appearance and produce strong thermoregulatory effects (Fig 1). By measuring reflectance values in the visible and near-infrared (NIR) spectra, Shi et al [7] have shown that the presence of triangular hairs greatly enhances light reflection. Sunlight is presumably deflected by a combination of scattering within the triangular hairs (Mie scattering) and total internal reflection (TIR) on their bottom face. Based on radiative heat transfer experiments, these authors showed with thermal camera images that hairs allow the workers to maintain lower body temperatures. The latter are achieved by minimizing energy absorption in the visible and NIR ranges of the spectrum, and by maximizing heat emissivity in mid-infrared (MIR) where the blackbody radiation spectrum of the ant’s body is at its maximum [7].

In this paper, we further investigate the geometrical optics underlying the high reflectivity of *C*. *bombycina* hairs. Using theoretical and empirical approaches, we demonstrate that the bright silver color of the ant *C*. *bombycina* stems from the TIR associated with the hairs that densely cover the ant body. First, we examined hair morphology using scanning and transmission electron microscopy. Second, using ray-tracing optical models, as well as bi-directional reflectance distribution function (BRDF) and attenuated total reflection (ATR) experiments, we provide evidence that the triangular shape of hairs allows TIR for a large range of incident angles. We also show that hairs are corrugated on their upper faces and that this enhances reflection properties, giving hairs an almost total specular reflectance beyond the critical angle at which TIR occurs, thus the silver color. Finally, we bring additional evidence on the effect of hairs as an adaptation to heat tolerance, by comparing reflectance and internal body heating rate between hairy workers and ‘shaved’ workers whose hairs were removed.

## Materials and Methods

Samples of *C*. *bombycina* were collected in the sand dunes of Erg Chebbi, Merzouga, in South-eastern Morocco (3108’45”N, 0359’55”W). The dunes are public areas and the silver ant is not an endangered or protected species; no permission was required to collect the samples. To compare the reflectance and heating rate between hairy, untreated workers and ‘shaved’ workers whose hairs were removed, ants were anesthetized by CO_2_ exposure and hairs covering the dorsal side of the abdomen were completely removed using a sharp scalpel blade

### Electron microscopy analyses of hairs morphology

Samples for scanning electron microscopy (SEM) were prepared by first removing the legs from workers, and then gluing the head, thorax and abdomen with silver paint on a metallic sample holder. The whole mount was coated in metal (20 nm of gold) in order to ease charge elimination, and placed in a JEOL InTouchScope JSM-6010LV microscope. For transmission electron microscopy (TEM), samples were prepared by embedding a piece of cuticle with hairs in an epoxy resin infiltrating the structure at a constant temperature of 35°C for 48 hours, and hardened at 60°C for 72 hours. Then, 90 nm-slices were cut perpendicular to the hairs’ axis and examined with a FEI Tecnai transmission electron microscope.

### Experimentally testing total internal reflection

To experimentally test the TIR model, we first measured the direction and spectral radiance of 2 mm^2^ of the abdomen of a *C*. *bombycina* worker using a viewing angle measurement system (Eldim EZContrast L80) that gives the full spectral bi-directional reflectance distribution function (BRDF). The detector gives accurate data for emissions ranging from 400 nm to 700 nm. This method allowed us to obtain a full BRDF image for each wavelength measured. In order to obtain reflectance values, spectral radiance was normalized with radiance of incident light. Reflectance of hairy workers was compared with that of shaved workers. Data were obtained for 3 individuals in each group; no significant intra-group difference was observed. Second, we experimentally tested attenuated total reflection (ATR) by placing hairs on a selectively absorbing surface, thereby greatly reducing TIR. Under ATR conditions, the light is transmitted rather than internally reflected in the hair [8]. Hairs were deposited flat face down on a copper grid (support for TEM samples) and examined with an optical microscope (Olympus BX61) in bright-field epi-illumination mode. The objective (x60) had a numerical aperture (0.85) large enough to illuminate a single hair under angles > 34.9°, which corresponds to conditions for observing TIR (see Results).

### Ant heating rate

A 0.075mm diameter thermocouple (K-type Thermocouple chromel–alumel, RS Components Ltd) connected to a digital thermometer (Digital Thermometer 206–3738, RS Components Ltd) was inserted through a small incision in the rectum of a freshly killed ant. The same position under the first tergite was always reached. We did not witness any bleeding or liquid loss that might have been a source of evaporative cooling. We let the abdomens reach room temperature (21°C) prior to measurements. The dorsal surface of each abdomen was exposed perpendicular to the light emitted by a solar simulator (ORIEL Corporation USA, No. 81172), away from any possible interfering surfaces. The device uses a 1000 W xenon arc lamp (power supply: ORIEL No. 68820). Wavelengths above 1100 nm were removed with a water filter to limit blackbody radiation measuring only the effect of visible and NIR radiation (ORIEL No. 61945). Temperature elevation of the abdomen was recorded every 5 sec for 90 sec to approximate temperature equilibrium. Measures were repeated 3 times per abdomen, on 7 hairy and 7 shaved individuals; only ants of same size were used. All experiments were performed under constant temperature in an air-conditioned room. Initial rate of heat gain K was estimated following [9], as K = (T_ex_*0.5)/t_1/2_), with T_ex_ being the excess temperature (*i*.*e*., the difference between ambient and sample temperature) and t_1/2_ corresponding to the heating half-time. Comparisons of mean temperatures between hairy and shaved *C*. *bombycina* were performed using unpaired *t*-tests, using *Graphad Prism6* software.

## Results

### Hair morphology

**Fig 2:**
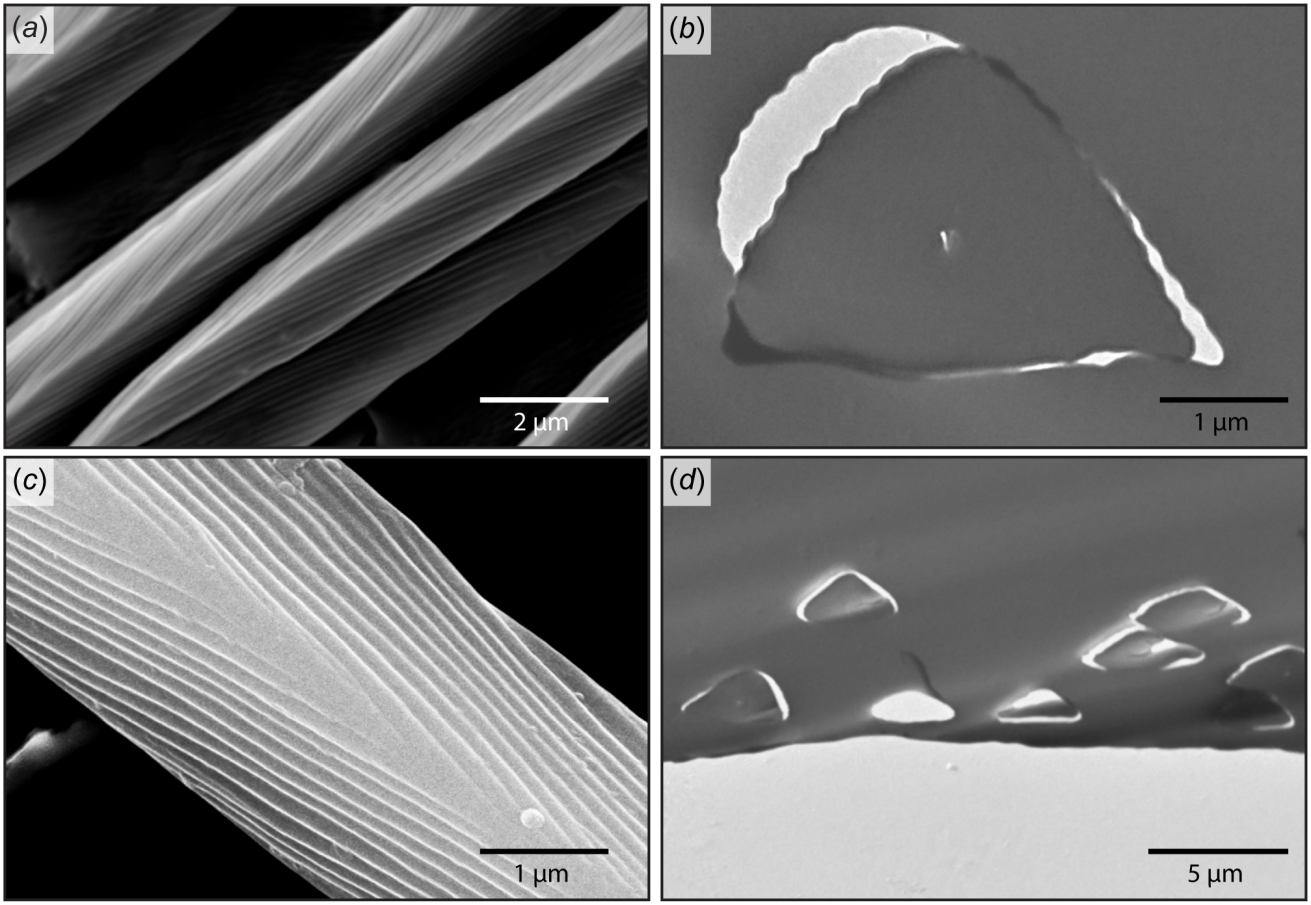
Electron microscopy images of the hairs covering the dorsal side of the body of *C*. *bombycina*. (a) Scanning electron microscopy (SEM) of the hairs covering the cuticle of ants. Hairs have a triangular shape and end in a sharp point. (b) Transmission electron microscopy (TEM) image of the cross-section of a hair. The two upper sides of the triangular shape are corrugated and the lower side (the triangle base) is flat. (c) Close up on the corrugations covering the two upper side of the triangular shape. (d) TEM image of hairs shows that they all adopt the same orientation, with their flat basal side parallel to each other and to the cuticle surface. The corrugated sides of the hairs are turned up as is clearly visible on image (a).

SEM analyses confirm that the hairs covering the cuticle assume a triangular cross-section (Fig 2a). Our detailed measurements reveal that they have a length of the order 200–300 μm, a width *w* = 3.5 μm and a height *h* = 2.4 μm. The angle at the top (± SD) is on average equal to 72 ± 2°, and the other angles (β) are both 54 ± 2° (*n* = 8). The two upper surfaces of each hair are corrugated by parallel grooves, while the bottom face (base) is not corrugated (Fig 2b). The corrugations on the upper sides are parallel 66 nm deep flutes separated from each other by 204 nm. The flutes run oblique to the longitudinal axis of the hairs (Fig 2c). TEM shows that the basal faces of the hairs are parallel to each other and to the cuticle surface of the ant (Fig 2d). The distance between the basal face and the cuticle surface varies from a few μm to 50 μm.

### Total internal reflection: prism model and experiments

#### Ray-tracing model

**Fig 3:**
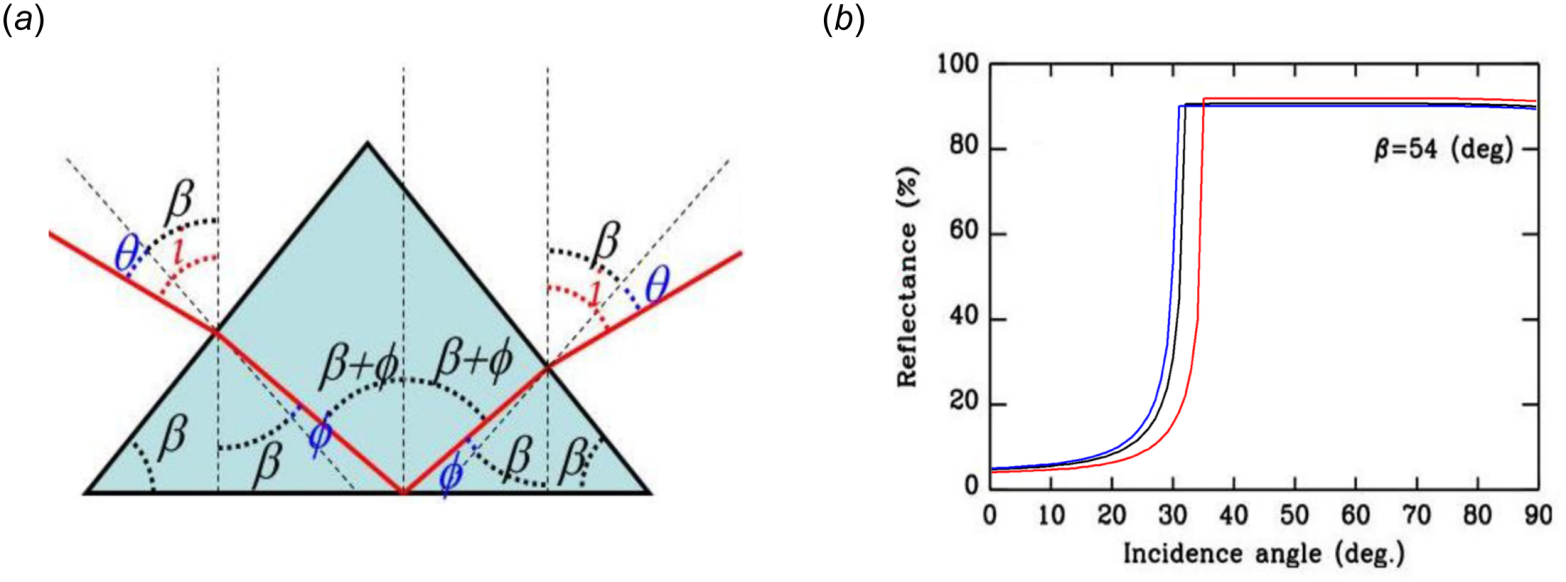
Total internal reflection prism model. (a) Ray-tracing model of TIR on the basal face of a triangular hair. The light enters through an upper face; it is totally reflected on the basal plane and exits through the opposite upper face. (b) Total reflectance of a hair (TIR and Mie scattering) as a function of the external incidence angle *i*, for an average chitin refractive index *n*_*chitin*_ = 1.56 (black), and for refractive indexes at 350 nm (*n*_*chitin/350*_ = 1.58; blue) and 800 nm (*n*_*chitin/800*_ = 1.51; red). For *n*_*chitin*_ = 1.56, the reflectance is low for sub-critical incidences *i* < 31.7°, then dramatically increases until *i* ≥ 34.9° at which angle total reflection occurs on the basal plane, parallel to the cuticle surface, at all visible wavelengths. A transfer-matrix model from wave optics results in a less steep transition from low to high reflectivity (data not shown).

The relatively small size of the hairs compared to the light wavelength does not hinder us from using geometric optics to model the light path. Fig 3a gives a schematic representation of a hair, with a light ray entering through one of the upper faces, totally reflected on the smooth basal plane, and exiting through the opposite upper face. After a sequence of transmission, reflection and, again, transmission, the light continues in the incident plane when neglecting weak reflections at the entrance and exit surfaces due to Mie scattering. Under these conditions, the incident and the emergence angles measured from the normal to the basal plane (*i*.*e*, the vertical) are equal (angle *i*, Fig 3a).

The reflection on the basal plane of the hair is total if the local incidence angle β + φ exceeds the critical angle *θc* given by

$$\text{sin}\theta_{c}=\frac{n_{air}}{n_{chitin}}$$ (1)

Considering a refractive index of chitin *n*_*chitin*_ = 1.56 [10,11] and a refractive index of air *n*_*air*_ = 1, Eq (1) gives a critical angle on the basal plane *θc* = 39.9°. To reach this critical angle, the external incidence angle of the light on the hair is

$$i=\beta -\text{arcsin}\left(\frac{n_{chitin}}{n_{air}}\text{sin}\left(\beta -\theta_{c}\right)\right)$$ (2)

Eq (2) gives the critical external incidence angle *i* = 31.7° with respect to the vertical, below which no TIR is possible. It should be noted, however, that the refractive index of the chitin is wavelength dependent [8,11–13] and directly affects the value of the critical external angle. Due to weak variation of the chitin refractive index in the visible range, *i* increases from 30.3° to 34.9° as wavelengths of incident light increases from 350 to 800 nm. That is, for each single hair of *C*. *bombycina*, incidences angles *i* larger than 34.9° should result in a very intense reflection at all visible wavelengths. This is illustrated in Fig 3b, which gives the calculated reflectance of a hair as a function of the external incidence angle *i*, for an average chitin refractive index of 1.56. In the high reflectance range, the overall reflection is not 100% because transmission through the upper faces of the prism is not perfect: a reflection in excess of about 5% removes intensity from the reflection on both the entry and exit faces. Still, more than 90% of the incident light is reflected. Thus, hairs are excellent mirrors for all external incidence angles *i* > 34.9°. Because reflection is directional for the whole visible wavelengths, this accounts for the silver sheen of the ant.

#### Influence of corrugations on reflectance

One noticeable feature of the hairs is the occurrence of corrugations on the entry and exit upper faces, contrasting with the flat basal face (Fig 2b). We explored the effect of such corrugations on reflectance. The spacing between two adjacent corrugations (*b* = 204 nm) is too short to produce diffraction. The effect of the corrugations is to slowly increase the effective refractive index when light enters the hairs, thereby acting as a kind of anti-reflection coating. This improves transmission of incident light through the upper surfaces of the hairs, and thereby increases the amount of light reflected by TIR.

To estimate the influence of corrugations on the light reflected by the entering face of a single hair, we compared the reflectance between a planar surface and a corrugated surface under normal incidence. For the planar surface, reflectance is calculated according to [14] as,

$$R={\left|\frac{n_{chitin}-n_{air}}{n_{chitin}+n_{air}}\right|}^{2}$$ (3)

**Fig 4:**
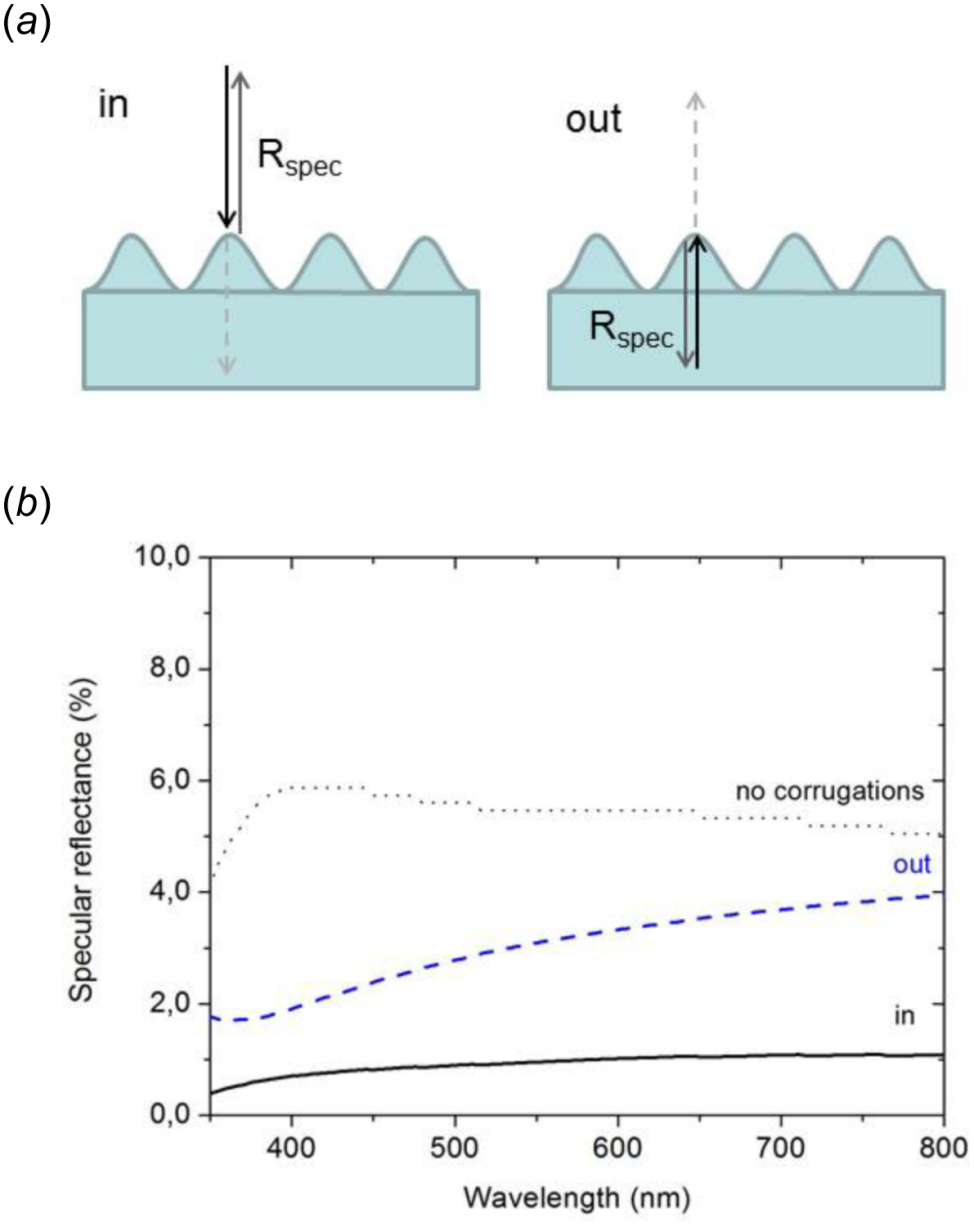
Influence of corrugations on the upper faces of the hairs on light reflection. (a) Schematic view of the corrugated chitin profile for light entering (in) and exiting (out) a hair. (b) Comparison of the specular reflectance between a flat chitin surface and a chitin surface corrugated by a sinusoidal profile, at normal incidence (see text). Corrugations significantly attenuate the reflection on both entry (in) and exit (out) of the light. This results in a global prism reflection ranging from 95% (800 nm) to 98% (350 nm) for incidence angles producing a total internal reflection. 64 monochromatic plane waves were used to reach the convergence of the transfer-matrix calculation.

The refractive index on the external side of the interface is *n*_*air*_ = 1. Taking into account that the refractive index of chitin depends on the wavelength of incident light, Eq (3) gives an average reflectance *R* = 5.4% (using a range of 350–800 nm) (Fig 4). For the corrugated surface, we approximated the corrugation profile by a sinusoidal function with amplitude of 66 nm and a pitch of 204 nm, in line with experimental values (see above). Using a scattering-matrix approach [15], we calculated the specular reflectance (*i*.*e*., incident angle = reflectance angle) of the sinusoidal profile. We considered a normal incidence for both the light entering and exiting the chitin. As shown Fig 4, corrugations considerably reduce the reflectance in the whole visible range. For the entering light, this reduction reaches a full order of magnitude, with a reflectance equal to *R* = 0.5%. Corrugations also reduce reflectance of exiting light, though to a lesser extent, with a specular reflectance varying from 2 to 4%.

Altogether, these results show that corrugations on the upper faces of a hair have two major optical consequences on the scattering: they increase substantially the amount of light entering each hair and the amount of light exiting the hair after the TIR. Ultimately, this enhances TIR on the basal face of the hair, thus the reflectance of the ant.

#### Influence of abdomen curvature on reflectance

The ellipsoidal shape of the abdomen leads to a wide distribution of incident light directions, most being in a non-perpendicular plane to the longitudinal axis of the hairs. By using a Fourier-transform based spectrometer, Shi et al. (2015) [7] showed that hairs enhance reflectivity over all angles and that reflectivity becomes particularly strong beyond 30°, which is consistent with our own TIR model presented Fig 3.

**Fig 5:**
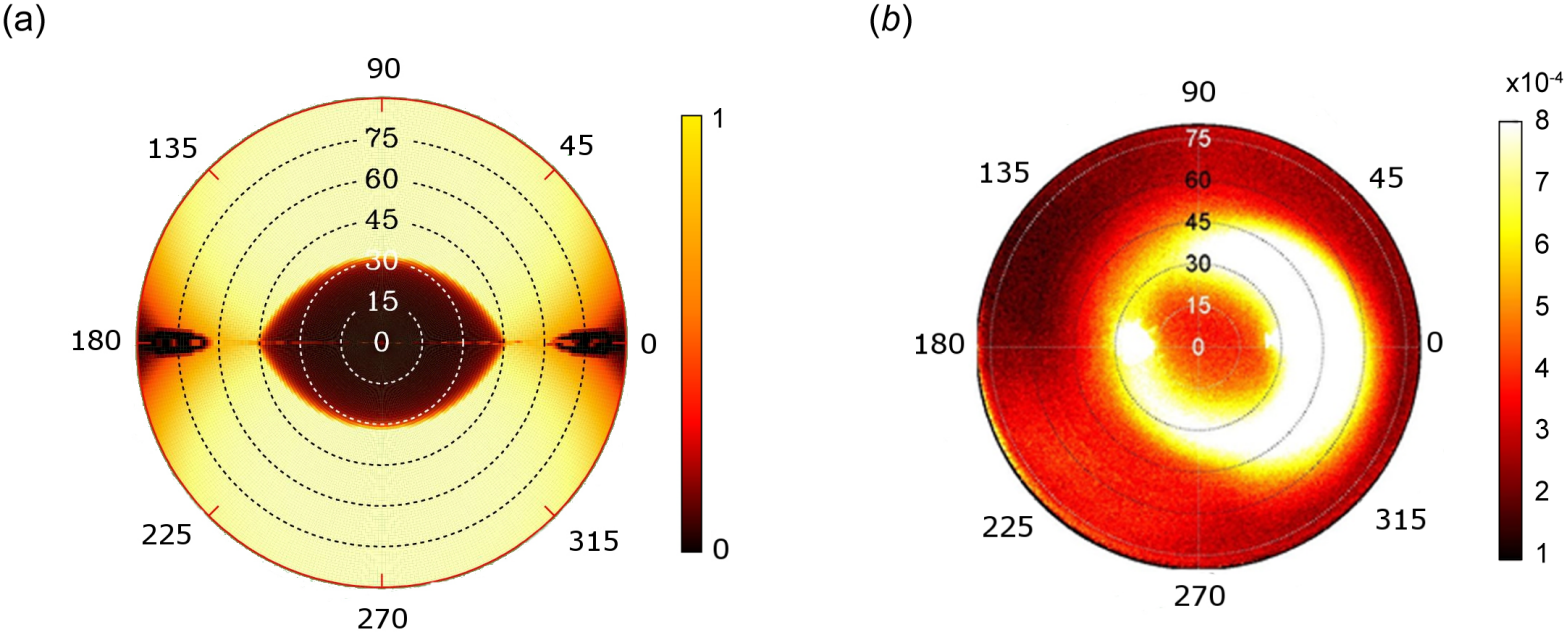
Spectral radiance maps of hairs in *C*. *bombycina*. Concentric circles correspond to the polar angles *θ* of the reflected light; counter-clockwise external angles are the azimuthal angles *φ* of the reflected light. (a) Calculation of the spectral radiance of a prism with triangular cross-section mimicking a single hair. The hair is oriented at an angle of *φ* = 0° and *θ* = 90° (azimuth 0–180 parallel to the axis of the hair). The reflection is total for all incidence angles, except at small incidence angles. (b) Experimental measure of the spectral radiance of a 2mm^2^ surface of a worker abdomen covered with hairs. The hairs are oriented perpendicularly to the incident plane (azimuth 90–270 parallel to the axis of the hairs). Shown are data for an incident wavelength of 498 nm; similar patterns are obtained for other wavelengths (400–700 nm).

To analyze the exact direction of the reflected light by the whole abdomen as a function of the incident angle of exposure, we summed the reflectance on a single hair over all incident directions [16]. In this situation, each incident direction is defined by a polar angle *θ* (*i*.*e*., the latitude of the incident light) and an azimuthal angle *φ* (*i*.*e*., the longitude of the incident light). Fig 5a gives the spectral radiance map on a hair for all incident directions. As shown, for all azimuthal angles TIR conditions are fulfilled for a wide range of polar angles. Consistent with the TIR conditions of the model (Fig 5a), spectral bi-directional reflectance distribution function (BRDF) analyses of the abdomen of *C*. *bombycina* workers show a ring of high reflectance for polar angles of approximately 30° to 60° (Fig 5b). For polar angles < 30° (*i*.*e*., the center of the intensity map) almost no reflection is detected, which is in agreement with a light passing through the hairs without TIR due to small incidence angles. The dark, external ring observed on Fig 5b for polar angles between 50° and 80° may result from two causes. First, it may stem from the viewing angle of the measurement system and its relatively low collection efficiency (65%) for high polar angles [17]. Second, the many hairs that are oriented parallel to the incident ray do not reflect the light at polar angles > 60°, as shown Fig 5a for a single hair. This particular orientation results in the hairs acting as waveguides. Asymmetry of measured radiance on Fig 5b is probably due to hairs being aligned on the body of the ants (Fig 1c), hence the effect of only a subsample of incidence angles could be detected.

#### Attenuated total reflection

**Fig 6:**
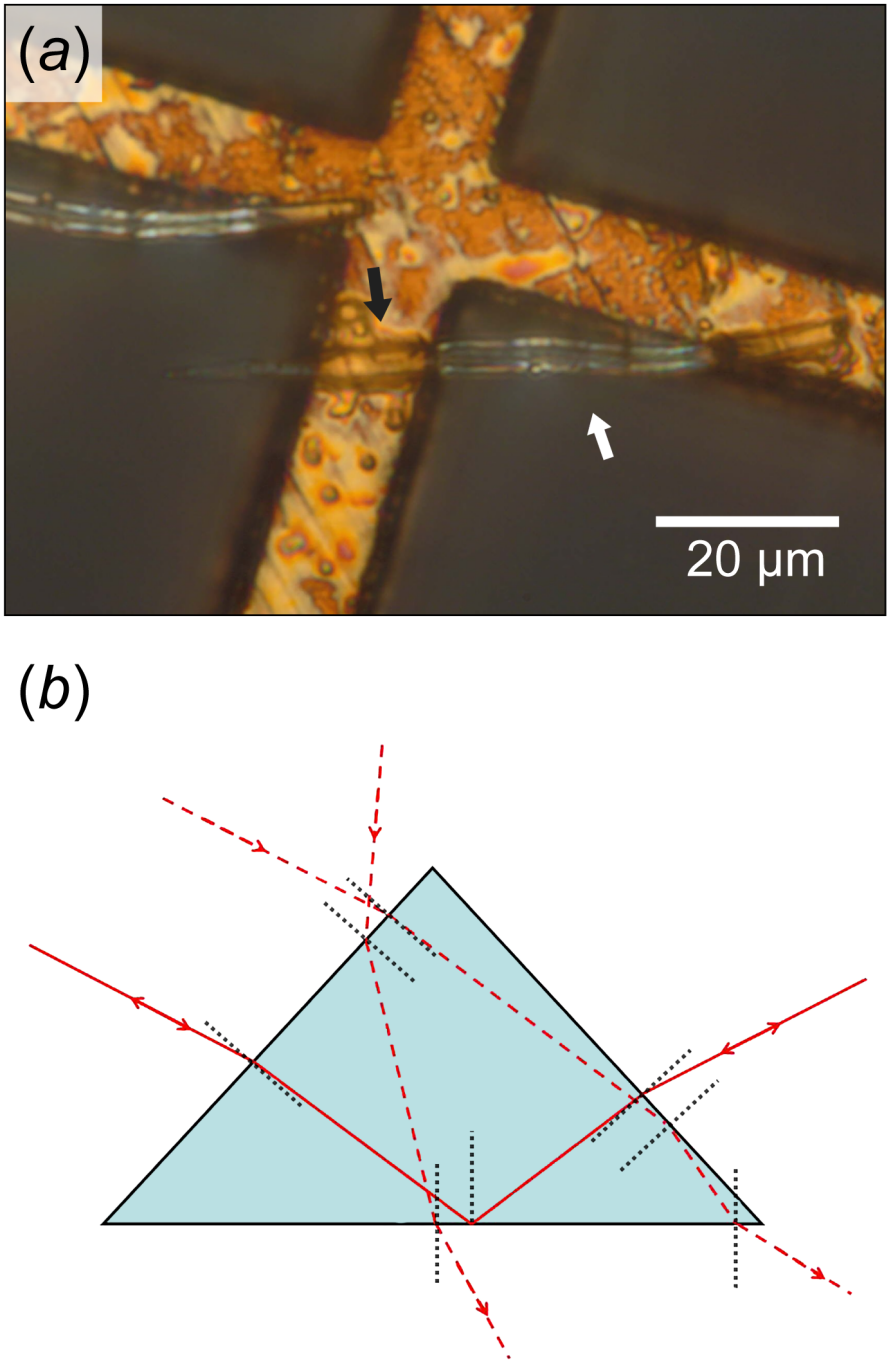
ATR experiment on ant hairs. The hairs are placed on an absorbing copper surface, with the bottom surface in contact with the copper. A part of each hair rests on the surface, whereas the other part is overhanging. Hairs are exposed to a spot of incident light large enough to hit both upper faces. (a) On the over-hanging part of the hair, TIR occurs on the basal surface so that the reflected light is observed on both upper faces of the hair (as shown by the two bright lines bordering it). In contrast, for the part of the hair in contact with the copper wire, TIR is attenuated by the proximity of the selectively absorbing surface (the two bright lines bordering the hair disappear). (b) Schematic diagram showing TIR on the basal face of a triangular hair (plain line); under TIR conditions, no light can exit from the top portions of the two upper faces of the hair (dotted lines).

Consistent with attenuated total reflection (ATR), TIR is dramatically reduced when hairs are placed on an absorbing surface of copper (Fig 6a). Highlights are observed on parts of the hair that overhang between wires, while parts in contact with the wire appear transparent so that the underlying copper surface can be seen. On the overhanging part, only the edges are bright; this is because TIR is effective on the basal plane only when light hits either side of the hair with an angle greater than 34.9° and/or when light enters the top of the hairs so that TIR occurs on the upper faces (Fig 6b).

#### Reflectance measurements

**Fig 7:**
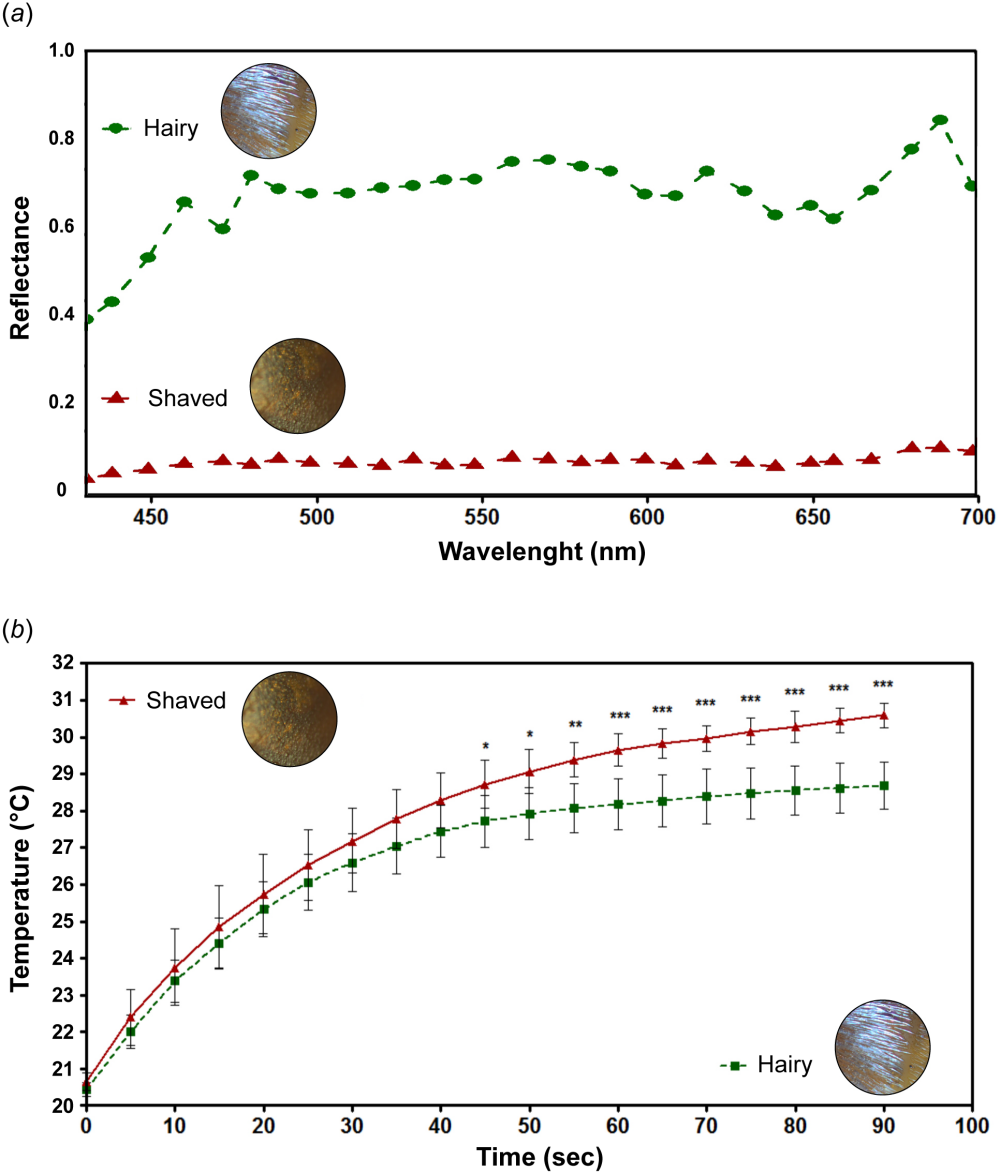
Reflectance and heating curves of hairy workers and shaved workers of *C*. *bombycina*. (a) Reflectance. TIR is induced on the ant abdomen with an incident light at 50° and azimuth 0. Reflectance is about 10-fold higher in the presence of hairs, for wavelengths ranging from 425 to 700 nm. (b) Heating curves. Abdomens are exposed for 90 seconds to the light emitted by a solar simulator whose visible spectral intensity is identical to the summer Saharan sun. Difference in excess temperature becomes significant after 45 seconds; after 90 seconds exposure, the difference in excess temperature between both conditions reaches 1.91°C. Unpaired Student *t*-test (*: *p* < 0.05, **: *p* < 0.01, ***: *p* < 0.001).

Comparison of the spectral radiance in the visible between hairy workers and shaved workers by BRDF measurements shows that the presence of hairs allows an almost constant 10-fold increase in radiance. As shown Fig 7a, mean reflectance between 425 and 700 nm is 0.61 for hairy ants and 0.07 for shaved ants. These results are consistent with Fig 3b showing very intense reflectance at all visible wavelengths for the given incidence (50°). They are also in line with [7] Fourier-transform based spectrometer measurements, showing a significant increase in light reflectance in the visible and near-infrared range of the spectrum on hair-covered regions of the body.

### Ant heating rate

We compared temperature elevation between shaved and hairy workers exposed to the light emitted by a solar simulator. The lamp intensity was calibrated according to the global irradiance of Saharan summer midday sun for a flat surface at sea level (Wilaya d'Adrar, Algeria; SMART95 software). Exposition to the solar simulator results in different heating curves between abdomens (Fig 7b). Averaged K values (± SD) are 0.27 ± 0.12 and 0.19 ± 0.03 for shaved and hairy abdomens, respectively (*t*-test, *p* = 0.15). Difference in excess temperature between shaved and hairy ants becomes significant after 45 sec exposition (1.00°C, *p* = 0.026); it is maximal after 90 seconds (1.91°C, *p*< 0.0001).

## Discussion

Our results show that total internal reflection in the hairs of the Saharan ant *C*. *bombycina* is responsible for its bright silver sheen. Each hair has the shape of a prism with a triangular cross-section within which the conditions for TIR are met for a wide range of incidence angles. In addition, our simulations indicate that corrugations on the entrance and exit faces of the hairs further increase reflectance across the whole visible wavelength range. For incidence angles ranging from 34.9° to 90°, the reflectance is very close to 100% for all azimuthal angles. For smaller incidence angles, light can leak through a hair. However, the light transmitted will be reflected by other hairs underneath. Together, these physical properties account for a strong mirror effect in the whole visible range, hence, the silver color of the ant. Consistent with this, ATR experiments show that the proximity of a metallic surface attenuates TIR, suppressing the silver color.

Our data show that the hairs covering the ant dorsal face reflect up to 10 times more light than shaved cuticles over the visible spectrum (425–700 nm). The values of reflectance obtained in the present study are similar to those reported by Shi et al. [7]. These authors measured reflectance values with a Fourier-transform based spectrometer allowing full visible and NIR spectral range, while our measures of radiance were performed by BRDF in the visible range only. The energy of direct sunlight is composed approximately of 40% visible and 50% NIR radiation (UV below 400 nm and IR beyond 1500 nm account for 2–4% and 6–8% of this energy, respectively;[18]). NIR radiation is therefore a major source of incoming energy that may greatly affect the thermal steady state of the ants. It was shown [7] that hairs also largely enhance NIR reflectance.

The thermoregulatory properties of the hairs coating stem from at least two effects [7]: (*i*) a reduction of solar energy intake by the mirror effect in the visible and NIR range of the spectra, which is further confirmed by our own results, and (*ii*) enhanced heat dissipation by radiation in the mid-IR where the black body radiation spectrum of the ant is at its maximum. By exposing individuals to a high-power xenon lamp to simulate the solar spectral distribution at the desert sand surface, these authors conducted an elegant set of thermodynamic experiments both in vacuum (to examine the effect of thermal radiation) and in air (to study the interplay of thermal radiation and convection). Thermal camera images gave evidence that hairy workers maintain significantly lower body temperatures than those with the hairs removed. Our heating rate experiments, based on direct measures of temperature in the body of ants, corroborate these results. They show that hairy workers exposed to solar simulated light experience a reduction in excess internal temperature up to 2°C compared with shaved workers. Even a small difference in body temperature may prove highly ecologically relevant for *C*. *bombycina*, as it can make the difference between life and death for such a small insect foraging so close to its upper critical thermal limit. If such a hair coating is an efficient way to prevent overheating, why hasn’t it evolved in more desert insects? Activity period of ectotherms is limited both by their lower and upper temperature tolerance. During the cooler Saharan season, dark-colored diurnal ectotherms will be at advantage by absorbing more direct solar energy, thereby increasing their daily activity. Outside this period, they typically hide during the hottest hours of the day [19]. In contrast, foraging activity in *C*. *bombycina* is restricted to a narrow thermal window (48–51°C) delimited by heat stress (upper limit) and predatory pressure by their main lizard predator (*Acanthodactylus dumerili*) (lower limit) [2]. Thus, their lower limit of activity is far above physiological demands, whereas their upper limit depends on their ability to withstand heat. Consequently, unlike other desert insects, *C*. *bombycina* would gain in reflecting incident sunlight all year round.

So far, TIR has been reported as limiting light extraction in fireflies [20], and as a side effect in superhydrophobic leaves immersed in water [21]. Nature often uses other (more complicated) methods to produce a bright metallic coloration [14,22]. Bragg mirrors, for instance, have been described in other Hymenoptera. This kind of mirror uses light-wave interference in a one-dimensional layered structure to produce a high specular reflection, but often over a limited range of wavelengths. A reflectance covering a broader range of wavelengths can be obtained with a stack of layers with graded-thickness, such as those encountered in some Scarabaeidae [23]. In beetles of the subfamily Rutelinae, the alternation of refractive index values in the multilayer is caused by the helical distribution of anisotropic chitin bars, in the form of a discrete Bouligand structure [24–26]. The large number of layers allows a highly efficient broadband reflectance that avoids iridescence. This structure is obviously much more complex than that found in the ant *C*. *bombycina*.

The triangular form of the hairs in the Saharan ant contrasts with the typical cylindrical shape [27,28] or flattened plate-like shape [29] of bristles documented in most arthropods. Depending on the species and their body position, the morphology of hairs has evolved to serve several functions including defense, locomotion, prey capture, pheromone dispersal, or temperature sensing [28,30,31]. The triangular shape that evolved in *C*. *bombycina* optimizes the backscattering of light and is responsible for the silver sheen of the ant. While reflection allows insects to gain less heat under direct sunlight [7,32], it could also serve other purposes such as camouflage [33] or communication [34]. Current studies indeed show that triangularly shaped hairs also occur in ground-dwelling ants from the rainforest, which are exposed to low insulation conditions (Simonis, unpubl. data). Therefore, several mechanisms might have been jointly implicated in positive selection for the reflective coating in the silver ant.
